# Multidrug-resistant VAP before and during the COVID-19 pandemic among hospitalized patients in a tertiary private hospital

**DOI:** 10.1017/ash.2023.470

**Published:** 2023-10-27

**Authors:** Alec Ann Alissa F. Aligui, Cybele Lara R. Abad

**Affiliations:** 1 The Medical City, Pasig City, Philippines; 2 Department Of Medicine, Division of Infectious Diseases, UP–Manila, Philippine General Hospital, Taft, Manila, Philippines

## Abstract

**Background:**

There is limited data on ventilator-associated pneumonia (VAP) and multidrug-resistant VAP (MDR VAP) among COVID-19 patients.

**Methods:**

A retrospective study in a single, tertiary, private hospital in the Philippines was conducted comparing the incidence, profile, and patient outcomes of MDR VAP during the pre-COVID-19 (2018–2019) and COVID-19 (2020–2021) periods.

**Results:**

In total, 80/362 (22%) patients developed VAP, 27/204 (33.75%) from pre-COVID-19 and 53/158 (66.25%) from the COVID-19 period, respectively. The majority were male [20/27 (74%) vs 34/53 (64%)], with a median age of 66 (range 35–90) and 67 (range 32–92) years in each period, respectively. Comorbidities were similar, except cardiovascular disease (14/27 vs 11/53 patients, *p*-value 0.005) and chronic lung disease (14/27 vs 9/53 patients, *p*-value 0.0012). VAP incidence density was 19.3/1000 and 27.8/1000 ventilator days (*p*-value 0.9819)]; median length of stay before VAP for pre- and COVID-19 periods was 17 and 10 days, respectively (*p*-value <0.0001). Extended-spectrum *β* lactamase (ESBL)-producing resistance increased significantly [1/27 (3.7%) pre-COVID-19 vs 15/53 (28.3%)] during COVID-19, while Carbapenem-resistant Enterobacteriaceae resistance was higher in the pre-COVID-19 period (15/27 [56%] vs 10/53 [19%]). Mortality was high in both periods at 93% and 83%, respectively. On multivariate analysis, only female gender was associated with MDR VAP in the COVID-19 period (OR =3.47, [CI 1.019, 11.824], *p*-value < 0.047).

**Conclusion:**

The frequency of VAP and MDR VAP increased during the COVID-19 period, despite a shorter duration of hospital stay. The mortality of VAP was extremely high. Factors associated with increased risk of VAP and COVID-19 need to be studied further, and preventive measures should be prioritized.

## Introduction

The COVID-19 pandemic continues around the world. Globally, there have been more than 600 million confirmed cases of COVID-19, including 6 million deaths as reported by the World Health Organization (WHO).^1^ Multidrug-resistant organism (MDRO) infections also continue to be prevalent, especially in healthcare settings. In other countries, the overall prevalence of MDRO is said to be 10.8 per 1,000 admissions.^2^ The COVID-19 pandemic is believed to have increased the prevalence of MDRO. The rise is attributed to multiple factors such as corticosteroid use, stay in long-term care facilities, immunosuppression, and uncontrolled antibiotic use, all having a possible significant effect on antimicrobial resistance.^3^

During the COVID-19 pandemic, a systematic review and meta-analysis of ventilator-associated pneumonia (VAP) among patients with COVID-19 infection was undertaken.^4^ Results showed that COVID-19 infected patients had a higher risk of developing VAP than patients without COVID-19 infection (OR 3.24; 95% CI 2.2–4.7; *P* = 0.015; I2 67.7%).^4^ Both bacterial co-infection and superinfection in critically ill COVID-19 patients are also common and are currently still being studied. Hospital-acquired infections including VAP are higher especially among patients with prolonged hospitalizations and among patients with underlying comorbidities.^5^ This study aimed to describe the incidence and clinical profile of patients with VAP and determine the predictors and outcomes of infected patients who developed multidrug-resistant (MDR) VAP during the pre-COVID and COVID-19 periods.

## Methodology

### Study population and sample

All patients in the intensive care unit who were mechanically ventilated during the study period from January 1, 2018, to December 31, 2021, were reviewed for possible inclusion. Only those patients with bacteriologically confirmed VAP were subsequently included in the cohort. For the COVID-19 period, only patients with confirmed COVID-19 via reverse transcription polymerase chain reaction (RT-PCR) testing and mechanically ventilated during the same admission were included. Excluded from the study were VAP patients with no microbiological confirmation and those who were transferred to another hospital or discharged against medical advice during their hospital stay.

### Study design and data collection

All data were retrospectively reviewed and collected by the study author (AAA) from 2018 to 2021 using available electronic data records from hospital information systems including IQVIA ArcusAir™, MIDAS, and Laboratory Information System (LIS) during the study period. Relevant information such as basic patient demographics (eg, age, gender, and comorbidities), laboratory information (eg, microbiologic data, complete blood count, electrolytes, and arterial blood gas), clinical assessments (eg, quick sepsis related organ failure assessment (qSOFA) and APACHE II in the first 48 h of admission), and data relevant to the primary outcome (eg, timing, incidence density, and therapies given for VAP) were recorded in a standard data collection form.

### Definition of terms

VAP was defined as pneumonia occurring >48 h after endotracheal intubation, using existing guidelines,^6^ and described as follows: a combination of radiologic, clinical, and microbiologic criteria in a ventilated patient for at least 48 h, with new or worsening infiltrates on imaging, with clinical signs such as fever, leukopenia, new-onset sputum or change in character, and worsening gas exchange.^6^ Common microbiologic techniques used in VAP diagnosis include retrieving samples from subsegmental bronchus via aspirating instilled sterile saline or bronchoalveolar lavage, the use of a double telescoping catheter with a metallic brush or protective specimen brush technique, and inserting a catheter and blindly aspirating secretions or blinded bronchial sampling.^7^ In this cohort, all respiratory samples were from endotracheal aspirates. Molecular methods for the diagnosis of nosocomial pneumonia such as point-of-care (eg, PCR tests)^8^ were unavailable and not utilized. Early-onset VAP was defined as VAP occurring within 4 days of hospitalization, while late-onset VAP was defined as VAP occurring on day 5 or later.^3^ VAP incidence density was calculated as # VAP/1000 ventilator days. Antibiotic or steroid use was defined as patients receiving at least one dose of antibiotic and/or one dose of systemic corticosteroid. MDROs were defined as being nonsusceptible to one or more agents in more than or equal to three antimicrobial categories.^6^ Extensively drug-resistant (XDR) VAP, a subset of MDR, was defined as nonsusceptible to one or more agents in all but less than two categories; pan-drug-resistant VAP was defined as being nonsusceptible to all microbial agents listed.^9^ Carbapenem resistance was defined as the resistance of an organism to at least one carbapenem drug. Extended-spectrum beta-lactamase (ESBL)-producing resistance was determined using phenotypic antibiotic susceptibility testing employed in the clinical microbiology laboratory. HL (High Level)-cephalosporinase was defined as microorganisms having high levels of cephalosporinase activity as determined by phenotypic antibiotic susceptibility testing.

### Statistical analysis

All data were analyzed using IBM-SPSS and STATA 17. A Chi-square test (for comparing independent proportions) was performed for the baseline demographic data, comparisons of proportions of MDR VAP, analysis by age class, hospital stay, comorbidities, and identification of microorganisms. Mann–Whitney test was done for comparison of medians from independent samples. Binary logistic regression was performed for the multivariate analysis of the variables with MDR VAP for both pre-COVID-19 and COVID-19 periods. A *p*-value of <0.05 was used as a cutoff for statistical significance.

## Results

### Clinical characteristics of patients

There were 362 mechanically ventilated patients in the intensive care unit (ICU) during the study period—204 in the pre-COVID-19 and 158 during the COVID-19 period. Overall, 80 patients developed VAP (80/362, 22%) and were included in this cohort—27 (33.7%) from the pre-COVID-19 and 53 (66.25%) from the COVID-19 period. Baseline demographics in both periods were similar, with most patients being male (20/27, 74% vs 34/53 64%) with a median age of 66 (range 35–90) and 67 (range 32–92) years, in the pre-COVID-19 and COVID-19 periods, respectively. Comorbidities were the same between the two periods, except for cardiovascular disease (*p*-value 0.005) and chronic lung disease (*p*-value 0.0012) which decreased significantly from pre-COVID-19 to the COVID-19 period. Other baseline characteristics were similar between the two periods (Table 1).

**Table 1.** Baseline demographic differences between pre-COVID-19 and COVID-19 periods*

Characteristics	Pre-COVID-19 period (2018–2019) (*n* = 27)	COVID-19 period (2020–2021) (*n* = 53)	*p*-Value
**Age, years**	66 (20)	67 (19)	0.847
**Gender**			
Female	7 (26%)	19 (36%)	0.3700
Male	20 (74%)	34 (64%)	
**BMI (kg/m** ^2^ **)**	26 (7)	26 (5)	0.128
**Smoker**			
Yes	14 (52%)	22 (42%)	0.2677
No	13 (48%)	31 (58%)	
**Comorbidities**			
None	2 (7%)	4 (8%)	0.8745
Diabetes mellitus	12 (44%)	23 (43%)	0.9324
Hypertension	20 (74%)	40 (75%)	0.9230
Cardiovascular disease	14 (52%)	11 (21%)	0.0050
Chronic lung disease	14 (52%)	9 (17%)	0.0012
Chronic kidney disease	4 (15%)	2 (4%)	0.0831
Chronic liver disease	1 (4%)	0 (0%)	0.1452
Neurologic	4 (15%)	1 (2%)	0.0255
Malignancy	1 (4%)	1 (2%)	0.6024
Human immunodeficiency virus (HIV) infection	0 (0%)	0 (0%)	1.0000
Thyroid disease	1 (4%)	1 (2%)	0.6024
**Previous history of antimicrobial use within 90 d (yes)**	9 (33%)	24 (46%)	0.2677
**Empiric antibiotics**			
Azithromycin	1 (4%)	4 (8%)	0.4998
Ceftazidime	1 (4%)	0 (0%)	0.1452
Ceftriaxone	5 (19%)	8 (15%)	0.6494
Clindamycin	2 (7%)	1 (2%)	0.2648
Meropenem	3 (11%)	6 (11%)	1.0000
Piperacillin-tazobactam	18 (67%)	38 (72%)	0.6456
**Corticosteroid use during admission (Yes)**	9 (33%)	53 (100%)	<0.0001
**Severity of illness score**			
qSOFA	2 (1)	1 (1)	0.660
APACHE-II	17 (7)	15 (5)	0.165
**Type of VAP**			
Early-onset	5 (19%)	8 (15%)	0.6494
Late-onset	22 (81%)	45 (85%)	
**Types of resistance**			
None	11 (41%)	15 (28%)	0.2430
Carbapenem-resistant	15 (56%)	10 (19%)	0.0008
XDR	0 (0%)	2 (4%)	0.2952
ESBL-producing	1 (4%)	15 (28%)	0.0115
HL cephalosporinase	0 (0%)	4 (8%)	0.1334
MRSA	0 (0%)	7 (13%)	0.0515
**Length of hospital stay prior to VAP (days)**	17 (9)	10 (6)	<0.0001
**Primary outcome**			
MDR VAP	15 (56%)	37 (70%)	0.2165
Non MDR VAP	12 (44%)	16 (30%)	
**Secondary outcome**			
Alive	2 (7%)	9 (17%)	0.2205
Death	25 (93%)	44 (83%)	
**VAP/1000 d**	19.3 (33.7%)	27.8 (66.25%)	0.9819

Note.

* Results are expressed in median (IQR) or *n* (%).

### Microorganisms, resistance patterns, and VAP treatment

**Figure 1.**
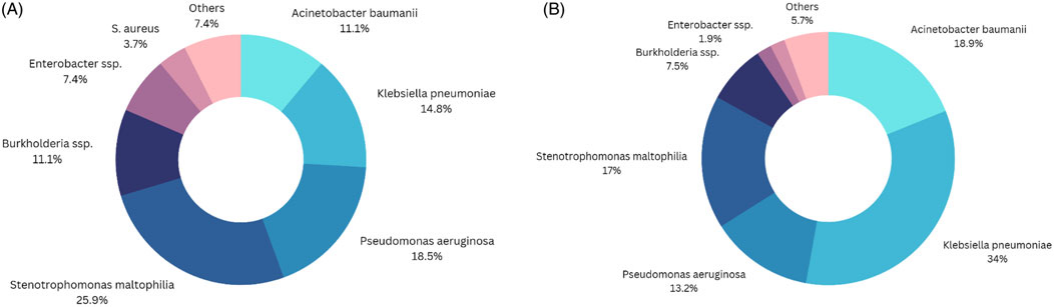
Identification of pathogens causing VAP A) pre-COVID-19 and B) during COVID-19.

The incidence of microorganisms was similar between pre-COVID-19 and COVID-19 periods. Among patients with VAP, *Klebsiella pneumoniae* was seen more frequently during the COVID-19 period (18/53, 34%) compared to the pre-COVID-19 period (4/27, 15%), although this was not statistically significant (*p*-value 0.0740). *Acinetobacter baumannii* (10/53 19% vs 3/27, 11%) (*p*-value 0.3627) was common in both study periods (Figure 1).

Similarly, among VAP patients included in this study, carbapenem resistance was higher during the pre-COVID-19 (15/27, 56%) than in the COVID-19 period (10/53 19%) (*p*-value 0.0008), while ESBL-producing resistance increased significantly from the pre-COVID-19 (1/27, 4%) to the COVID-19 period (15/53 28%) (*p*-value 0.0115). Additional drug resistant organisms, such as Methicillin-resistant Staphylococcus aureus (MRSA), also increased from 0% in pre-COVID-19 to 13% (7/53) during COVID-19 (*p*-value 0.0515).

In both periods, the most frequently used empiric antibiotic drug was piperacillin-tazobactam (18/27, 67% vs 38/53, 72%) followed by ceftriaxone (5/27, 19% vs 8/53, 15%) and meropenem (3/27, 11% vs 6/53, 11%). Corticosteroid use in the form of dexamethasone was significantly higher in the COVID-19 period compared to the pre-COVID-19 period [53/53, 100% vs 9/27, 33% (*p*-value <0.0001)].

### Outcomes

The incidence of VAP during pre-COVID-19 and COVID-19 periods in our study was 13.2% and 33.5%, respectively, with the incidence density at 19.3/1,000 days and 27.8/1,000 days, respectively (*p*-value 0.9819). MDR VAP increased from pre-COVID-19 to COVID-19 periods (15/27, 56% vs 37/53, 70%; *p*-value 0.2165) but was not statistically significant. The median length of stay prior to documented VAP for pre-COVID-19 and COVID-19 periods was 17 (range 5–54) and 10 (range 2–27) days, respectively (*p*-value <0.0001). The majority developed late-onset VAP, with 81% (22/27) and 85% (45/53) during the pre-COVID-19 and COVID-19 periods, respectively. Mortality from VAP was high in both periods, with (25/27) 93% of patients with VAP dying in the pre-COVID-19 period and (44/53) 83% in the COVID-19 period.

### Risk factors associated with MDR VAP

All variables analyzed for the pre-COVID-19 period were not significantly associated with MDR VAP (Table 2). However, in this period, patients with underlying lung disease were 1.6 times more likely to develop MDR VAP than those without lung disease (OR 1.6 [CI 0.346, 7.401]). During the COVID-19 period, only female gender appeared to be associated with an increased risk of MDR VAP (OR 3.4 [CI 1.019, 11.8], *p*-value 0.047) (Table 2).

**Table 2.** Multivariate analysis on the association of the different variables with MDR VAP

	Pre-COVID-19 (*n* = 27)			COVID-19 (*n* = 53)		
Variable	OR	95% CI	*p*-Value	OR	95% CI	*p*-Value
Age	0.956	[0.899, 1.017]	0.153	1.004	[0.959, 1.052]	0.858
Hospital stay (numeric)	0.974	[0.912, 1.040]	0.435	1.018	[0.891, 1.163]	0.789
Hospital stay: less than 16 d	0.357	[0.074, 1.719]	0.199	0.344	[0.038, 3.124]	0.343
Hospital stay: 16–20 d	0.400	[0.062, 2.568]	0.334	0.427	[0.046, 3.890]	0.455
Hospital stay: more than 20 d	0.667	[0.123, 3.617]	0.638	0.000	[0.000, –]	1.000
Comorbidity	0.000	[0.000, –]	0.999	1.324	[0.127, 13.785]	0.815
Cardiovascular disease	0.476	[0.102, 2.230]	0.346	1.429	[0.353, 5.788]	0.617
Lung disease	1.600	[0.346, 7.401]	0.548	0.612	[0.112, 3.334]	0.570
Gender: male	2.500	[0.389, 16.049]	0.334	0.288	[0.085, 0.981]	0.047
Gender: female	0.400	[0.062, 2.568]	0.334	3.471	[1.019, 11.824]	0.047
BMI	0.943	[0.775, 1.148]	0.560	0.935	[0.790, 1.106]	0.433
Smoker	0.875	[1.191, 3.999]	0.863	0.535	[0.155, 1.846]	0.322
qSOFA	0.680	[0.317, 1.459]	0.322	1.106	[0.440, 2.780]	0.830
APACHEII	0.987	[0.880, 1.108]	0.825	0.862	[0.748, 0.993]	0.039
Previous use of antibiotics	0.500	[0.095, 2.645]	0.415	1.250	[0.384, 4.068]	0.711
Use of corticosteroid	1.964	[0.388, 9.933]	0.414	0.000	[0.000, –]	1.000
Type of VAP: early onset	0.800	0.111, 5.772	0.825	0.738	0.132, 4.123	0.729

Note. *p* > 0.05, not significant; *p* ≤ 0.05, significant. Test used: binary logistic regression analysis.

## Discussion

Our study on VAP during two different periods highlighted the following—first, VAP and MDR VAP frequency increased in the COVID-19 period. Second, the onset of VAP after hospitalization was faster during the COVID-19 period. Third, we were unable to identify risk factors associated with MDR VAP, except for female gender, in the COVID-19 period. Fourth, Gram-negative organisms were frequent, with ESBL-producing organisms more common in the COVID-19 period, and finally, the mortality of patients with VAP was extremely high.

Around 22% of ventilated patients in our ICU (80/362) developed VAP during the period of study. That approximately one in five patients develop VAP is not unusual and is similar to other studies. An observational cohort study on VAP in Nepal showed a similar incidence rate of 20% with an incidence density of 16.45 cases per 1,000 ventilator days.^10^ In addition, a systematic review and meta-analysis also reported an overall cumulative VAP incidence of 23.5%.^11^ In our study cohort, patients with COVID-19 infection were more likely to develop VAP compared to patients without COVID-19. The incidence density of VAP during the COVID-19 period was almost double that of the pre-COVID-19 period (19.3 vs 27.8/1,000 ventilator days). This was similarly reported by another study which showed an increased frequency of VAP during the COVID-19 pandemic at 48% compared to 13% prior to COVID-19 with an overall incidence density of 28/1,000 vs 13/1,000 (*p* = 0.009).^12^ This increased frequency of VAP among COVID-19 patients is perhaps linked to their predilection for bacterial co-infection due to structural lung damage from COVID-19 infection^13^; moreover, the addition of immunosuppressive agents such as corticosteroids and immunomodulatory medications may also predispose patients to bacterial superinfection in the form of VAP.^14^

We also hypothesize that the global shortage of healthcare supplies during these early stages of the COVID-19 pandemic could have potentially contributed to the higher VAP rate during the COVID-19 period. Although our institution created adaptations to both hospital structure and staff in response to surge capacity,^15^ the lack of personal protective equipment such as N-95 masks and gowns, and other critical supplies such as ventilator tubings, which were either recycled or used for longer than recommended, may have increased the potential for cross-contamination. The workforce was also in short supply, and many nurses and staff who were diverted to the ICU had limited training in infection control or were unfamiliar with working in the critical care setting. In addition, compliance with the VAP bundle, which was at 84.6% during the pre-pandemic period,^16^ was not monitored closely during the pandemic and may have declined further, especially since certain bundle elements (eg, head of bed elevation, readiness to extubate, and oral care) may have been more difficult to assess among critically ill COVID-19 patients who were in isolation and who were either pronated or shielded under an aerosol box.

In our cohort, COVID-19 infection was associated with a faster median onset of VAP compared to non-COVID-19 patients (10 vs 17 days). Increased length of hospital stay is usually reported as a risk factor associated with increased VAP frequency.^17^ However, patients with COVID-19 infection developed VAP despite a shorter duration of hospital stay; this may indicate a sicker cohort of patients (eg, rapid progression of COVID-19 from severe to critical), or perhaps an impaired immune system that specifically predisposed COVID-19 patients to bacterial infection,^18^ explaining the faster onset of VAP among these patients. COVID-19 is known to be associated with cytokine storm and immune dysregulation. Increased cytokine expressions of interleukin-6 (IL-6), interleukin-8 (IL-8), and monocyte chemoattactant protein-1 (MCP-1) are said to be a markers for bacterial and fungal superinfections.^19^ Knowing this, it is not surprising that critically ill patients with COVID-19-associated immune dysregulation are predisposed to superinfection such as VAP.^20,21^

In both pre-COVID-19 and COVID-19 studies,^5,22^ gender has been identified as an independent risk factor for VAP, with an increase in frequency among males. This is in contrast to our study, where female patients had a higher risk of MDR VAP compared to males. Our finding was similar to another study where there was increased ICU mortality among females (20.5%) compared to males (14.45) (*p* = 0.113); on logistic regression, female gender was associated with an increased ICU mortality risk [OR 1.775 (CI 1.029–3.062, *p* = 0.039)].^23^ However, the relationship of gender with COVID-19 and the subsequent risk of infection such as VAP is controversial. There is heterogeneity of data on the role of gender in COVID-19, and several factors are proposed to influence outcomes, including variations in hormonal and immune system responses,^23^ as well as social behaviors such as smoking.^24^ In our cohort, there were no significant differences in baseline demographics of males and females in both pre-COVID-19 and COVID-19 periods, including comorbidities. Thus, no conclusion can be made regarding the role of gender as a risk factor for MDR VAP among COVID-19 patients, but it warrants further exploration.

The most frequently isolated microorganisms during the COVID-19 period in our cohort were Gram-negative organisms (*K. pneumoniae* and *Acinetobacter baumannii)*. This is similar to other studies,^25,28^ including in the local setting, where *K. pneumoniae* was commonly isolated among COVID-19 patients admitted in a tertiary-level government hospital.^26^ However, in our cohort, there was an increase in ESBL-producing *K. pneumoniae* during the COVID-19 period compared to the pre-COVID-19 period. The reasons for this are likely multifactorial, but frequent use of broad-spectrum antimicrobials such as piperacillin-tazobactam and ceftriaxone may have contributed to the emergence of ESBL-producing resistance.^27^ MRSA frequency was also much higher in the COVID-19 period, which is not surprising since MRSA has been reported to be one of the common causes of bacterial co-infection in patients with COVID-19.^29^ In another study, a higher rate of MRSA infection was seen among COVID-19 patients compared to non-COVID-19 patients (65% vs 27.5%).^30^ Surprisingly, however, the frequency of carbapenem resistance in our study was lower in the COVID-19 period, contrary to other studies. A possible explanation for this might be due to the local microbial ecology where there is a higher baseline incidence of *K. pneumoniae* with ESBL-producing resistance. In our institution, microorganisms with carbapenem resistance usually emerge later, after prolonged hospitalization and extensive antimicrobial pressure. Another potential explanation is the small number of patients included in this study which limits inference and generalization. In general, multidrug resistance in COVID-19 patients is likely increased due to overuse of empiric antibiotics, even among those patients with unestablished bacterial pneumonia.^31^

VAP has been associated with a significant increase in the 28-day mortality rate among COVID-19 patients.^31^ This high mortality rate is associated with the increased inflammatory state which predisposes them to immunosuppression.^32^ Disease severity among COVID-19 patients with VAP increases due to an impaired adaptive immune system, especially in patients developing acute respiratory distress syndrome (ARDS).^33^ However, the overall mortality rate in this study (83% and 93%) was high regardless of the study period and higher than reported by other studies, which ranged from 19.4% to 69.2%.^34–37^ Mortality rate is often influenced by the presence of MDRO infection, septic shock^36^ and a higher SOFA score,^38^ all of which were present in this cohort of patients. Other specific risk factors may have contributed to our higher mortality rates, and these need to be identified and studied further.

This study is not without limitations. It is from a single institution and is retrospective, and the small sample size may have underestimated the incidence of MDR VAP and risk factor association. Given the limited number of non-COVID-19 patients admitted during the COVID-19 period, a direct comparison of VAP rates of two different populations within the same time frame was not possible. The results may not be generalizable to another setting. A multicenter study is necessary to further strengthen the factors associated with VAP and to provide more information regarding VAP in other health settings. Nevertheless, it is one of the few studies in the region to characterize and compare VAP among patients in the pre-COVID-19 and COVID-19 periods.

Overall findings in this study highlight that VAP mortality is high. Critically ill COVID-19 patients appear to be at high risk of VAP and MDR VAP compared to non-COVID-19 patients. VAP onset appears to be earlier for COVID-19 patients and should be suspected when patients worsen clinically. Gram-negative pathogens are frequently responsible for VAP and should be considered when deciding empiric therapy. Risk factors associated with MDR VAP should be explored further.
